# A metabolic switch in the TCA cycle: a regulator of intestinal stem cell fate during tissue regeneration

**DOI:** 10.1038/s41392-025-02414-5

**Published:** 2025-10-06

**Authors:** Dong Gil You, Jae Hyung Park

**Affiliations:** 1 Department of Chemical Engineering and Biotechnology, Tech University of Korea, Siheung, Republic of Korea; 2 School of Chemical Engineering, College of Engineering, Sungkyunkwan University (SKKU), Suwon, Republic of Korea; 3 Department of Health Sciences and Technology, SAIHST, Sungkyunkwan University, Suwon, Republic of Korea

**Keywords:** Regeneration, Metabolic engineering

**Fig. 1:**
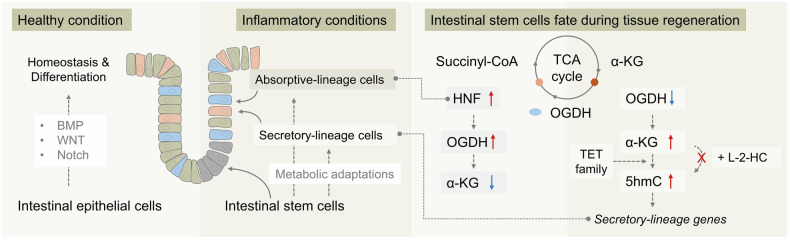
Metabolic adaptations direct ISC fate during tissue regeneration. ISCs reside at the base of the small intestinal crypts, where they exhibit self-renewal capacity and give rise to all differentiated epithelial lineages. While transcriptional regulation has long been recognized as the primary mechanism governing homeostasis and lineage commitment, this study suggests that mitochondrial metabolism—particularly the TCA cycle—also plays a critical role in fate determination. ISCs give rise to progenitor cells that differentiate into either absorptive or secretory lineages, a process in which the mitochondrial metabolite α-KG serves as a key regulatory signal. In absorptive cells, high expression of OGDH, driven by the transcription factor HNF4, leads to α-KG depletion. In contrast, secretory progenitors exhibit low OGDH expression, resulting in intracellular accumulation of α-KG. Consequently, the TET family catalyzes the formation of 5 hmC, thereby promoting the expression of genes associated with the secretory lineage. Moreover, L-2-HG, a competitive inhibitor of α-KG, suppresses secretory marker gene expression, highlighting a potential strategy for metabolic targeting in precision medicine

In a recent study published in Nature, Chaves-Perez et al. identified a key metabolic switch that determines cell fate during tissue regeneration.^1^ Their findings revealed that intestinal stem cells (ISCs) differentiate into mature absorptive and secretory lineages by regulating cellular levels of α-ketoglutarate (α-KG), a mitochondrial metabolite involved in a regenerative tricarboxylic acid (TCA) cycle (Fig. 1).

Stem cells in the intestinal crypt play a critical role in maintaining the structural and functional integrity of the intestinal wall by continuously supplying new cells and tightly regulating the processes of self-renewal and differentiation.^2^ During homeostasis, a subset of progeny differentiates into absorptive enterocytes, which increase the villus surface area to enhance nutrient uptake. In contrast, others commit to secretory lineages—including goblet cells, Paneth cells, and enteroendocrine cells—that produce mucus, antimicrobial peptides, and regulatory hormones essential for intestinal immune function. However, under conditions of injury or chronic inflammation, the secretory cell population becomes depleted, leading to a disruption in epithelial lineage balance and consequent impairment of intestinal homeostasis.^3^

The homeostasis and differentiation of intestinal epithelial cells are tightly regulated by key signaling pathways, including bone morphogenic protein (BMP), WNT, and Notch, in conjunction with a network of lineage-specific transcription factors.^4^ Despite the extensive characterization of these regulatory cues, the impact of metabolic state alterations on epithelial regeneration and lineage specification remains insufficiently understood, particularly within the context of complex tissue environments affected by inflammatory disorders such as Crohn’s disease and ulcerative colitis, where homeostatic regeneration is impaired. Therefore, determining the role of metabolic pathways in governing cell fate decisions is a critical, yet unresolved, question in regenerative biology.

Chaves-Perez et al. investigated the role of the mitochondrial TCA cycle and provided direct in vivo evidence that metabolic adaptation functions as a pivotal determinant of ISC fate. Mitochondria are traditionally regarded as cellular powerhouses responsible for ATP production through the catabolism of proteins, lipids, and carbohydrates. They also generate key intermediates that fuel biosynthetic processes. However, recent perspectives have repositioned mitochondria as dynamic regulators of cellular function and lineage commitment rather than passive energy suppliers.^5^ In this context, the authors showed that α-KG, a TCA cycle intermediate, serves as a critical metabolic signal that directs ISC differentiation in vivo. Moreover, the authors’ findings suggest that α-KG, beyond its role as a cofactor for chromatin-modifying enzymes, directly regulates lineage specification during tissue regeneration, expanding its known function in epigenetic control of stem and progenitor cells.

Distinct expression patterns of TCA cycle enzymes were observed across intestinal epithelial lineages. In absorptive enterocytes, the expression of 2-oxoglutarate dehydrogenase (OGDH)—which catalyzes the conversion of α-KG to succinyl-CoA—was markedly elevated. In contrast, secretory progenitors, including goblet, Paneth, and enteroendocrine cells, exhibited low OGDH expression, resulting in intracellular accumulation of α-KG. These lineage-specific metabolic profiles not only reflect differential bioenergetic demands but also contribute to instructive cues that guide stem cell fate decisions. Using intestinal organoids and in vivo mouse models, the authors showed that genetic suppression of OGDH or exogenous administration of cell-permeable α-KG promoted differentiation toward secretory lineages, while compromising the survival and expansion of absorptive progeny. These findings indicate that high OGDH activity is essential for absorptive lineage maintenance, whereas secretory lineage specification is favored under conditions of reduced OGDH expression and α-KG accumulation.

α-KG functions as both a metabolic intermediate and an epigenetic regulator, acting as a cofactor for histone and DNA demethylases that govern transcriptional programs. In particular, the ten-eleven translocation (TET) family of dioxygenases, which catalyzes the oxidation of 5-methylcytosine (5mC) to 5-hydroxymethylcytosine (5hmC), is strictly dependent on α-KG to facilitate DNA demethylation and subsequent gene activation. In this study, TET protein levels were comparable between absorptive and secretory progenitor cells. However, secretory precursors, characterized by α-KG accumulation, exhibited a marked increase in 5 hmC levels accompanied by the upregulation of secretory lineage genes. Conversely, treatment with L-2-hydroxyglutarate (L-2-HG)—a competitive inhibitor of α-KG—led to reduced 5 hmC levels and suppression of secretory marker gene expression. These findings suggest that α-KG accumulation orchestrates epigenetic reprogramming via TET-mediated DNA demethylation, thereby promoting commitment to the secretory lineage.

This study further identifies hepatocyte nuclear factor 4 (HNF4) as a lineage-specific transcriptional regulator that links metabolic state to cell fate decisions. In absorptive enterocytes, HNF4 directly induces the expression of OGDH, thereby promoting the oxidative metabolism of α-KG to support the elevated ATP production and biosynthetic requirements characteristic of this lineage. The resulting depletion of α-KG limits TET enzyme activity, sustaining DNA methylation and reinforcing transcriptional programs that favor absorptive lineage differentiation. These findings highlight the coordinated interplay between transcriptional and metabolic networks in directing epithelial cell fate.

Notably, the therapeutic relevance of this metabolic mechanism was supported by a study using a murine model of colitis, which is characterized by impaired secretory cell maturation and deficient epithelial regeneration. Inhibition of OGDH or supplementation with exogenous α-KG restored secretory lineage differentiation and enhanced epithelial repair, highlighting the potential of targeting α-KG-centered metabolic pathways to promote mucosal regeneration under inflammatory conditions. However, further comprehensive research is needed to evaluate the translatability of these murine findings to human intestinal biology and to determine whether similar metabolic adaptation mechanisms regulate regeneration across different tissue types or injury contexts. Moreover, although targeting key metabolic pathways holds therapeutic potential, their pleiotropic roles in diverse cellular processes raise concerns about unintended effects, emphasizing the need for tissue- and context-specific strategies.

In conclusion, this study provides compelling evidence that α-KG functions as a metabolic signaling molecule governing ISC fate, thereby suggesting that metabolic pathways can act as upstream regulators of gene expression and tissue regeneration, beyond their classical roles in energy production and biosynthesis. By delineating the mechanistic link between cellular metabolism, epigenetic remodeling, and lineage specification, the study uncovers a novel regulatory axis with broad implications for regenerative medicine and the treatment of inflammatory disorders. Future investigations into the cell-type-specific regulatory mechanisms of the HNF4 gene and the context-dependent actions of α-KG will be critical for refining tissue-selective regenerative strategies.
